# Novel biomarkers in the saliva of healthy young males and females in a randomized crossover study on sedentary time: An exploratory analysis

**DOI:** 10.1371/journal.pone.0308838

**Published:** 2024-08-20

**Authors:** Emmeline Meens Miller, Nicholas O’Rourke, Michael Jeffrey, Julia Green-Johnson, Shilpa Dogra

**Affiliations:** 1 Faculty of Health Sciences (Kinesiology), University of Ontario Institute of Technology, Oshawa, ON, Canada; 2 Faculty of Science (Biology), University of Ontario Institute of Technology, Oshawa, ON, Canada; UNAM Facultad de Estudios Superiores Zaragoza: Universidad Nacional Autonoma de Mexico Facultad de Estudios Superiores Zaragoza, MEXICO

## Abstract

Several known biomarkers have been used to understand the physiological responses of humans to various short and long-term interventions such as exercise or dietary interventions. However, little exploratory work has been conducted to identify novel biomarkers in human saliva that could enable non-invasive physiological research to understand acute responses to interventions such as reducing sedentary time. The purpose of this study was to identify novel biomarkers in the saliva (cytokines, growth factors and vascular factors) that respond to prolonged (4 hours) and interrupted sitting (4 hours of sitting interrupted by 3 minutes of walking at 60% of maximal heart rate every 27 minutes) in young, healthy males and females. We also sought to determine whether responsive biomarkers would differ by sex. Participants (n = 24, 21.2 ± 2.2 years, 50% female) completed a prolonged sitting (PS) session and an interrupted sitting (IS) session in random order. Individual saliva samples were pooled into a male sample and a female sample to identify responsive biomarkers using a human cytokine antibody membrane array (42 targets). Several novel biomarkers were responsive in both sexes (e.g., IL-8, Angiogenin, VEGF, and EGF), in females only (e.g., TNF-α and IL-13), and in males only (e.g., IL-3, RANTES, and IL-12p40/p70). Importantly, several biomarkers appear to be responsive to the 4-hour prolonged and interrupted sitting sessions (e.g., TNF-α, IL-8, IL-3, RANTES, EGF, Angiogenin, and VEGF). This work highlights new directions for researchers aiming to investigate the effect of short-term or acute interventions on different physiological pathways using non-invasive methods. Our work clearly indicates that human saliva samples can provide a wealth of insight into physiological responses, and that a number of biomarkers can be used to understand changes induced by acute interventions such as interrupting prolonged sitting.

## Introduction

Biomarkers can be measured using different bodily fluids (e.g., blood, saliva, and urine), and muscle biopsies. Previously, blood and muscle biopsies were considered the “gold standard” in the analysis of biomarkers in exercise physiology [1]. However, these techniques are invasive, creating challenges for data collection and participant recruitment. Furthermore, while serum and plasma have been used widely, and therefore have established efficacy and accuracy [2,3], a consequence of drawing blood is that acute inflammation can occur due to the stress induced within the participant during blood collection. As such, invasive methods may be problematic for studies aiming to understand the physiological changes observed from an acute stressor or intervention. Alternatively, saliva is a safe and non-invasive method, that does not pose the same risk of inducing inflammation during collection [2,3].

Saliva sampling has been shown to be feasible and insightful for the measurement of cytokine responses to stressors. It is also more stable than blood, and therefore requires less preparation for analysis while providing a large number of analytes [4]. A review by Szabo et al. found that 17 cytokines found in the saliva were reported as detectable and responsive to acute stressors across multiple studies [3]. Saliva has also been shown to be an accurate method of sampling and to be applicable across multiple clinical settings [5,6]. However, few experimental studies assessing acute responses have used saliva. This is also the case in the area of sedentary physiology.

Sedentary behavior is defined as any waking behavior that has an energy expenditure of ≤ 1.5 metabolic equivalents, while in a sitting, reclining, or lying posture [7]. Data indicates that Canadians spend over 10 hours per day engaging in sedentary activities [8]. Accumulating high volumes of sedentary time is associated with increased risk of mortality [9–12], cardiovascular disease, and metabolic diseases such as diabetes [13–17]. It has also been shown to negatively affect cardiometabolic biomarkers [12], vascular function [18–21] and pro-inflammatory biomarkers [22,23]. Importantly, a growing body of research indicates that acute bouts of sitting can lead to negative physiological outcomes [24–26]. Two studies from our laboratory have investigated the acute response of Interleukin (IL)-8, a pro-inflammatory cytokine, in the saliva in this context. Both studies used a prolonged sitting session (4 hours of sitting) and compared this to an interrupted sitting session where 4 hours of sitting was interrupted by physical activity every 30–60 minutes [27,28]. Analysis revealed that salivary IL-8 levels increased during the prolonged sitting session, and the response was either attenuated or abolished during the interrupted session depending on the intensity of the physical activity. While IL-8 appears to be a promising marker, it is not clear if other markers, particularly those related to cardiovascular or pro-inflammatory pathways, would respond to such protocols.

Another gap in this area relates to sex differences. In our previous work, sex differences were noted when comparing IL-8 responses to prolonged and interrupted sitting [28]. Sex-differences have been noted in observational studies and in intervention studies in response to a variety of short and longer-term interventions related to movement and diet. These responses have been observed for vascular function markers (flow mediated dilation), and biomarkers such as IL-6, IL-8, Fibrinogen, CRP, glucose, cortisol, and RANTES [24,28–33]. It has been hypothesized that these differences are due to hormones, physical activity levels, inflammatory responses, body mass index, chronic disease states, diet composition, or cardiovascular fitness [28,29,31–34]. Whether these sex differences are also detected using salivary measures is unclear.

Saliva sampling is a promising method of assessing changes in biomarkers in response to acute interventions. However, no studies to our knowledge have investigated the array of biomarkers that are either detectable or responsive in the saliva in acute experimental studies such as our prolonged and interrupted sitting protocols. As such, we conducted an exploratory study aimed at identifying 1) detectable biomarkers present in the saliva and 2) biomarkers that respond to prolonged and interrupted sitting.

## Methods

**Fig 1:**
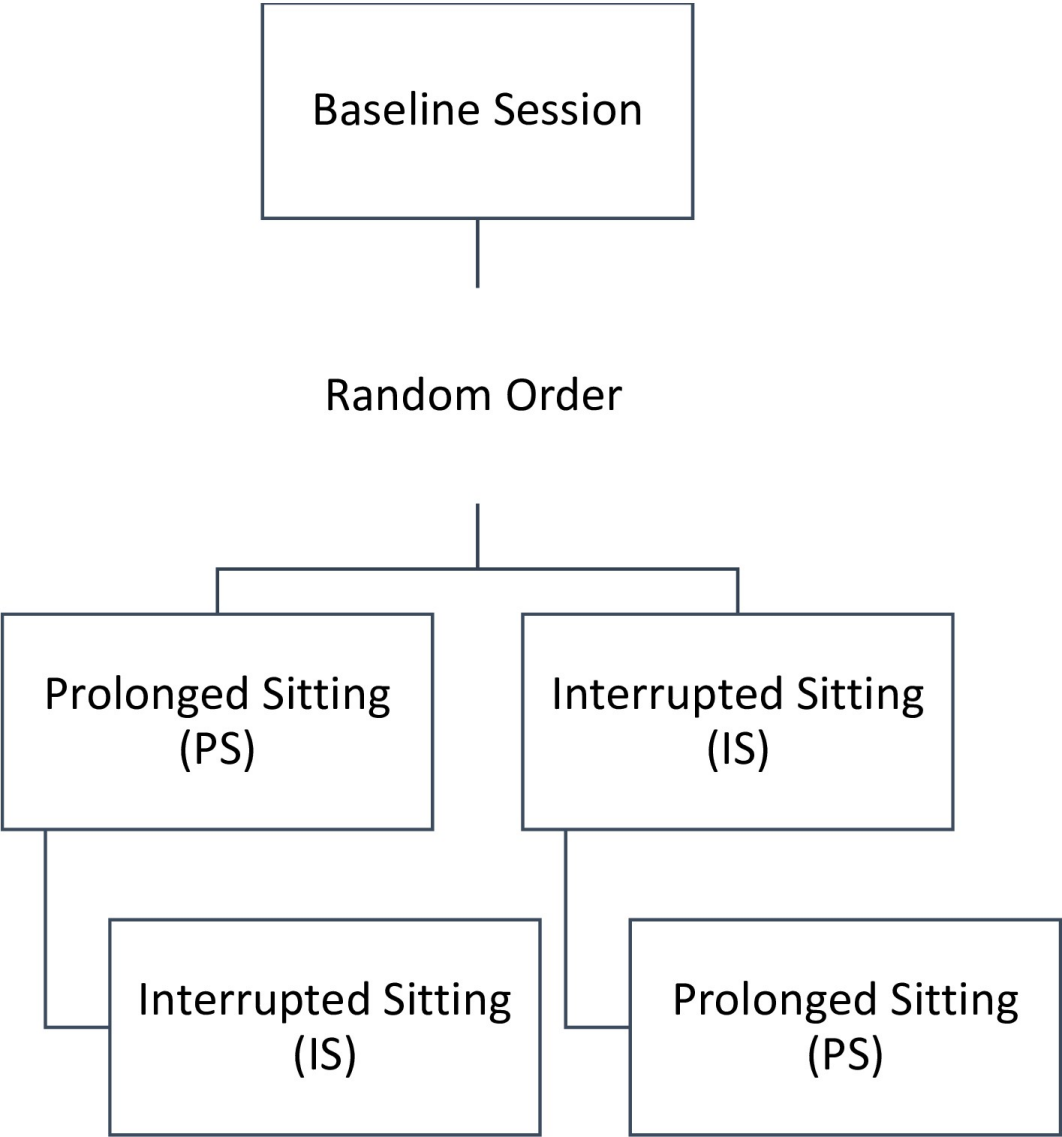
Experimental design. Visual representation of the experimental design. Participants completed a baseline session and then completed the Prolonged Sitting (PS) or Interrupted Sitting (IS) sessions in random order.

*Study Design*: A randomized crossover design was used (**Fig 1**). Participants were randomly allocated to complete either the **Prolonged Sitting (PS)** session or the **Interrupted Sitting (IS)** session first using a number generator, with balanced randomization. Sessions took place at least one week apart.

### Participants

Eligible participants were males and females between the ages of 18–30 years, with a body mass index (BMI) of <30 kg/m^2^, who were non-smokers. Individuals were excluded if they had an existing respiratory, cardiovascular, or metabolic condition, an acute infection, were pregnant, were taking any medication that would influence their inflammatory or exercise response, had an acute injury, or had recent dental surgery and/or any known oral disease. All participants provided written informed consent prior to participation in the study. This study was approved by the Research Ethics Board at Ontario Tech University (REB #16473). Data collection began on September 22, 2021 and ended on April 27, 2022.

Due to the exploratory and novel nature of this research, as well as the sex-based pooled analysis approach, a sample size calculation was not possible as there was no one effect size. Literature on microarray-based analyses to examine effects of exercise [35–37] and of microarray-based analyses of saliva [27,38,39] have used a sample size of n = 10–24; based on this we chose a sample size of 24 participants. Additionally, a previous study from our laboratory using the same interruption protocol, investigating salivary and plasma IL-8, completed a sample size calculation that indicated an n = 21 was sufficient [28].

### Protocols

Participants completed three laboratory sessions: 1) baseline session, 2) PS, and 3) IS.

#### Session 1: Baseline session

Resting HR, resting blood pressure (A&D Medical Digital Blood Pressure Monitor, Model UA-767FAM, A&D Engineering, Inc. San Jose, CA, USA), height (cm), and body mass (kg) (Detecto Weight Beam Eye-Level, Webb City Missouri) were measured. An incremental to maximal exercise test using a stepwise protocol was performed on a treadmill (Trackmaster, FullVision, Newton, KS). Participants were fitted with a portable HR monitor (Polar Electro Oy, Professorintie 5, FI-90440 Kempele, Finland) for continuous measurement of HR. A metabolic cart was used for breath-by-breath gas analysis (Parvo Medics 2400, USA). Concentrations of expired O_2_ and CO_2_ were analyzed, and ventilation was measured. Test termination criteria included a Respiratory Exchange Ratio (RER) >1.15, HR ± 10 beats per minute of age-predicated maximal HR (220-age), a plateau in oxygen uptake (VO_2_), or volitional fatigue. The highest HR recorded during the test was used for HRmax. VO_2_max was calculated as the highest VO_2_ that was attained during the test. After finding the peak VO_2_, VO_2_max was calculated as a mean of ±5 breaths, including this value.

#### Session 2 and 3: Prolonged and interrupted sitting sessions

**Fig 2:**
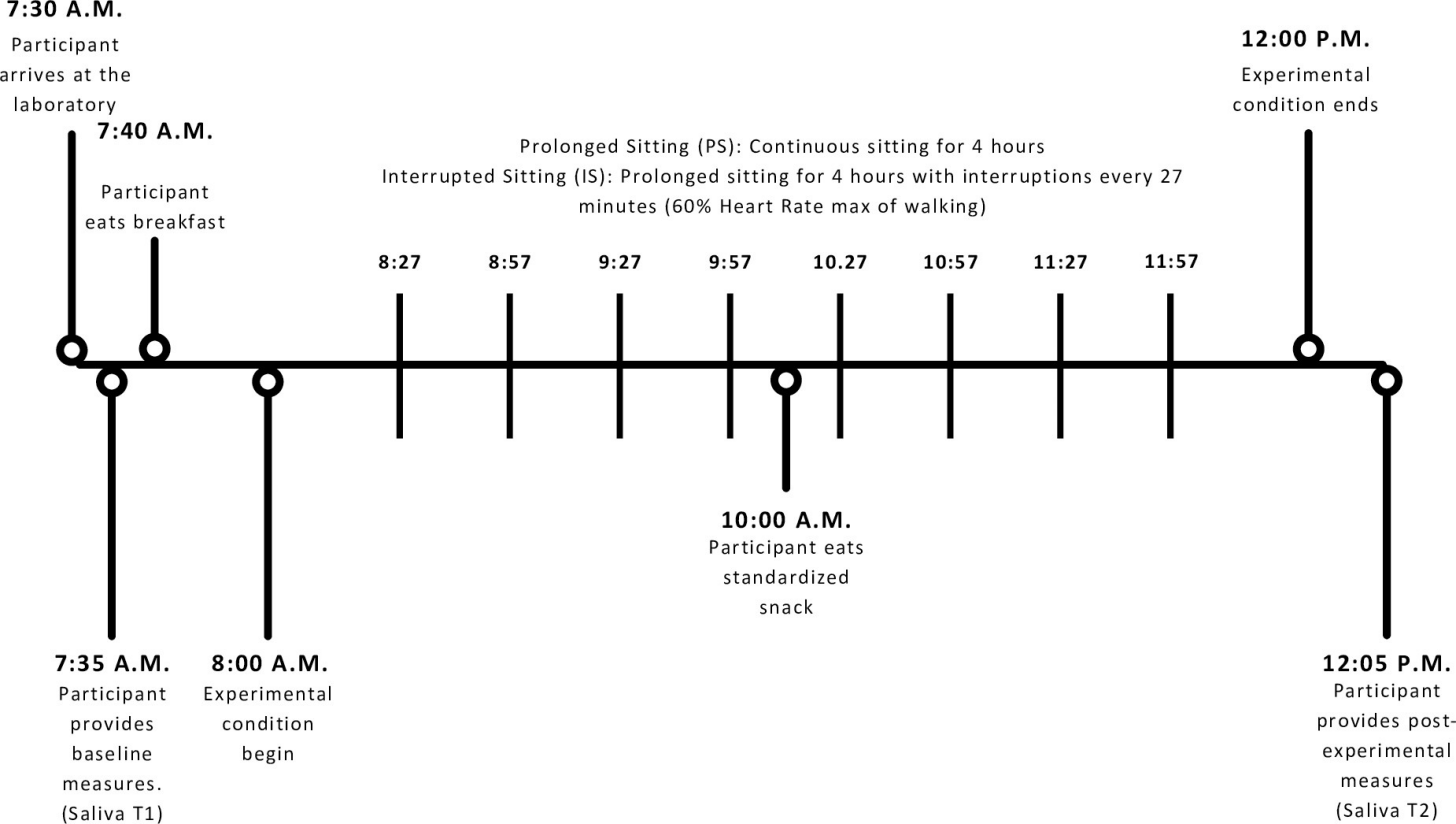
Prolonged and interrupted sitting sessions timeline. Visual representation of the Prolonged Sitting (PS) and Interrupted Sitting (IS) protocols. This timeline provides an outline of the sample collection timing and interruption timings.

These sessions were conducted in random order. Data collection was conducted from October 2021 to February 2022. For the PS, participants were seated continuously for four hours (**Fig 2**). For the IS, the four-hour prolonged session was interrupted with three-minute activity interruptions (60% Heart Rate (HR)max) every 27 minutes (at times: 27, 57, 87, 117, 147, 177, 207, and 237 minutes), for a total of eight interruptions. The four-hour duration was chosen as it best mimics a typical sitting pattern. Participants were instructed to perform as little lower limb movement as possible, however, they were permitted to use their upper limbs for the study duration. Participants were instructed to arrive fasted and drink 1L of water prior to arrival. They were provided a standardized breakfast and snack during the sessions (590 Calories; Fat: 6.5g, Carbohydrates: 127g, Protein: 11.1g).

#### Saliva collection and analysis

Whole saliva samples were taken upon arrival (T1) and at the end (T2) of each of the PS and IS sessions. Participants were instructed to refrain from alcohol, smoking, strenuous exercise, and the use of anti-inflammatory medications in the 24-hours prior to the session. They were also instructed to refrain from caffeine/stimulants, supplements, or mouthwash on the day of the session.

Saliva samples were collected using oral swabs (Salimetrics SalivaBio, Salimetrics LLC, State College, PA, USA). The participant was instructed to rinse their mouth with water to remove any debris or particulates and then the swab was placed under the tongue or against the cheek for five minutes and then immediately centrifuged (VWR Clinical 2000, Germany) at 4000 rpm for five minutes allowing collection of 1.5–3.0 mL of saliva, which was subsequently stored at -80°C. Saliva sample collection timing was matched between the two conditions, samples at T1 were collected between 7:30–8:00 a.m., while saliva samples at T2 were collected between 12:00–12:30 p.m. for both the PS and IS protocols. Samples were thawed on the day of analysis and were centrifuged for 15 minutes at 1500 x g at 4°C to remove mucins and particulate matter that could potentially interfere with antibody binding. Samples of individual participants were then pooled into 8 samples by sex (female and male), session (PS and IS), and time (T1 (before sitting) and T2 (end of 4-hour session)).

**Fig 3:**
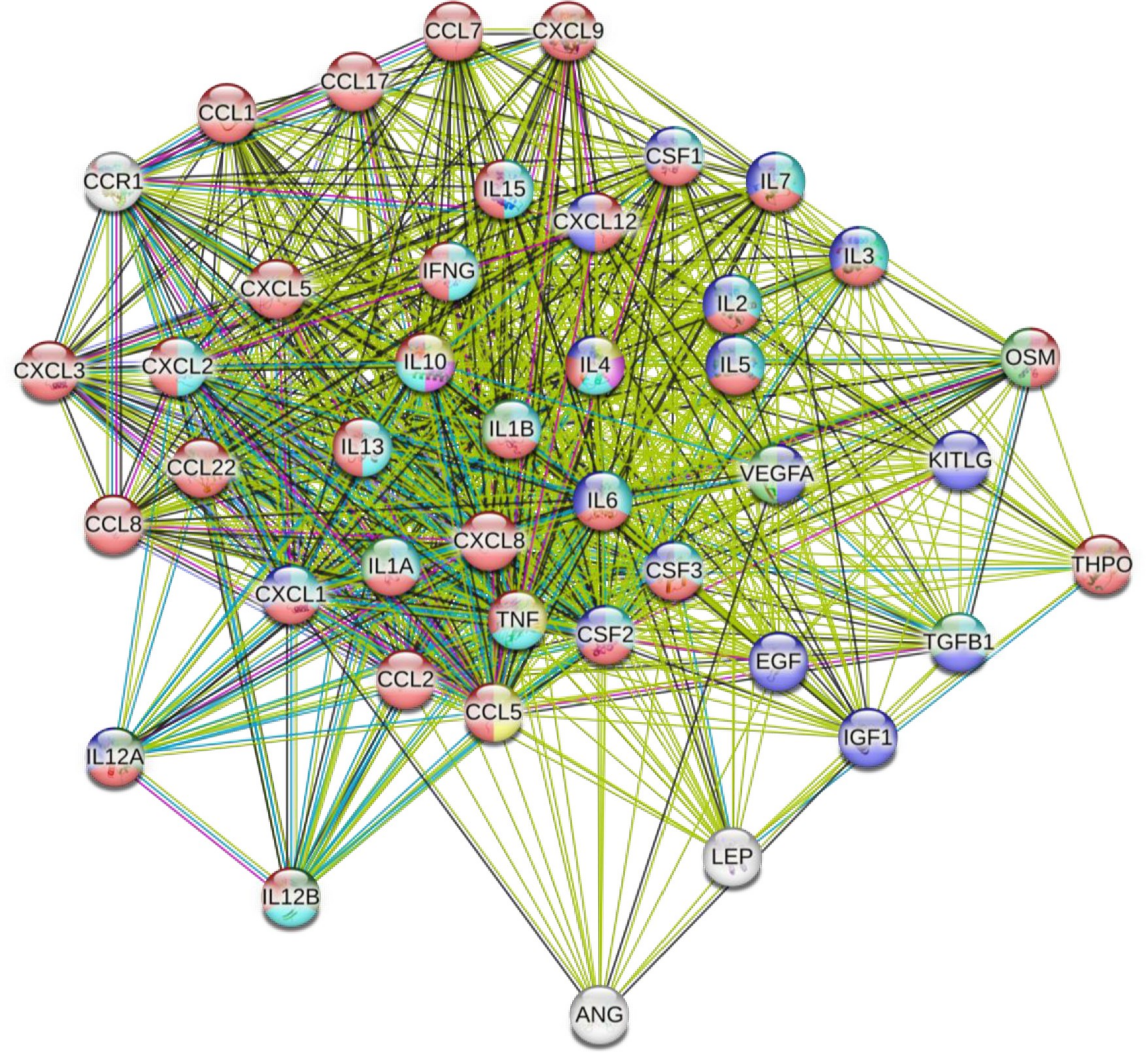
Physiological pathways and connections between the 42-Targets of the antibody array. Connections between 42 cytokines and growth factors with potential interactions are displayed using STRING v11.5 analysis. The 42 targets are connected using green (text mining), grey (co-expression), pale blue (protein homology), royal blue (gene co-occurrence), red (gene fusions), lime green (gene neighbourhood), blue (from curated databases), and pink (experimentally determined). Nodes are coloured to represent different functional pathways and to identify the biomarker as either a cytokine (red), growth factor (blue), or mitogen (light green). IL-3, IL-4, Il-5, CSF1 (MCSF), OSM (Oncostatin M), CSF2 (GM-CSF), THPO (Thrombopoietin), IL-10, IL-12A/IL-12B (IL-12), KITLG (SCF), IL-1A (IL-1⍺), IL-1B (IL-1β), IFNG (IFN-γ), LEP (Leptin), CCL2 (MCP-1), IL-2, CCL8 (MCP-2), CCL7 (MCP-3), CXCL5 (ENA-78), CCL22 (MDC), CXCL9 (MIG), CXCL8 (IL-8), CXCL1/CXCL2/CXCL3 (GRO), CCL5 (RANTES), CXCL1 (GRO-⍺), CCL1 (I-309), CCL12 (SDF-1), TNF (TNF-⍺/TNF-β), IL-6, IL-7, IL-13, Il-15, EGF, ANG (Angiogenin), CSF3 (GCSF), VEGFA (VEGF), CCR1 (MIP-1δ), IGF-1, CCL17 (Thymus and Activation Regulated Chemokine (TARC)), TGF-Β1 (TGF-β1).

Total salivary protein concentrations of the 8 pooled samples were determined using the Coomassie PLUS 138 Protein Assay Reagent (Thermo Fisher Scientific, MA, USA) prior to sample analysis. Cytokines, including several chemokines, growth factors and vascular/endothelial factors within the saliva samples were quantified using Human Cytokine Antibody Array Membranes following manufacturer’s protocols (Abcam, Catalog # ab133997). The full list of biomarkers analyzed by the microarray kit and their alternate names are presented in **S1 Table.** This particular array was chosen based on the pathways indicated in **Fig 3**, and their importance for sedentary physiology.

For analysis of samples, 250 μg of total protein (allowing for standardization across samples) from the pooled samples were loaded onto the membranes and subsequently incubated overnight at 4°C under gentle rotation. Following the overnight incubation, an extra wash was performed prior to loading the biotin-conjugated anti-cytokine antibody onto the membranes. Next, a wash was performed and the HRP (Horseradish Peroxidase)-conjugated Streptavidin was added to the membrane and incubated for 2-hours at room temperature. Lastly, a detection buffer containing HRP substrate was added and incubated for 2-minutes at room temperature and then chemiluminescence was measured. Chemiluminescence detection and semi-quantitative determination of cytokine expression within the different samples was performed using a LiCor C-DiGit® Blot Scanner and Image Studio™ imaging software. To calculate relative cytokine and growth factor expression levels, the summed signal density of each spot was background corrected and normalized to the positive controls across all membranes. Therefore, the **mean spot pixel density** is normalized to the positive controls across all membranes and indicates relative expression levels, despite not having a standard concentration-based unit of measurement. The concentration of targets detected by the Abcam ab133997 membrane array is typically in the pg/mL range, based on information provided by the manufacturer.

#### Data analysis

Descriptive statistics (Means ± SD) were performed on sample characteristics. Human cytokine antibody array results from the pooled samples were compiled. As this study took on an exploratory approach, similar to previous exploratory work [40], samples were pooled into male and female subgroups. As such, samples were not used to detect individual level marker concentrations, and therefore no statistical analysis can be performed to compare changes, instead the detection of biomarkers and their responsiveness to the different conditions can be visually inspected using average pixel density.

The Search Tool for the Retrieval of Interacting Genes/Proteins database (STRING v11.5, https://string-db.org/) was used to examine relationships between responsive and detectable cytokines and growth factors. Pathways were generated through the use of Ingenuity Pathways Analysis (IPA QIAGEN Inc.) to examine the different pathways the biomarkers are involved in (https://www.qiagenbioinformatics.com/products/ingenuity-pathway-analysis) [41,42].

## Results

Participant characteristics (n = 24, mean 21.1 years ± 2.2, 50% female) can be found in Table 1. Of the 42 targets, 26 were detectable. Several cytokines and growth factors were below detectable levels in the saliva (IL-2, MCP-2, TNF-β, MCP-3, IL-5, IL-6, IL-7, MIP-1δ, IL-10, I-309, IL-1α, TARC, IFN-γ, G-CSF, GM-CSF, and Leptin). Some cytokines and growth factors were only detectable in females (TNF-α, Thrombopoietin (only IS-T2), and IL-13 (only PS-T2)), while some cytokines and growth factors were only detectable in males (IL-3, IL-4 (only IS-T2), GRO, RANTES, GRO-α/CXCL-1, IL-12, SCF, PDGF-BB, and SDF-1/CXCL-12).

**Table 1:** Participant characteristics.

	Female (n = 12)	Male (n = 12)
**Age (years)**	20.9 ± 1.8	21.3 ± 2.5
Body Mass Index (kg / m^2^)	24.4 ± 2.1	25 ± 3.6
Physical Activity (minutes · week ^-1^)	154.2 ± 74.9	158.8 ± 189.6
Maximal Aerobic Capacity–VO_2max_ (mL · kg^-1^ · min^-2^)	37.2 ± 4.9	45.6 ± 8.5

**Fig 4:**
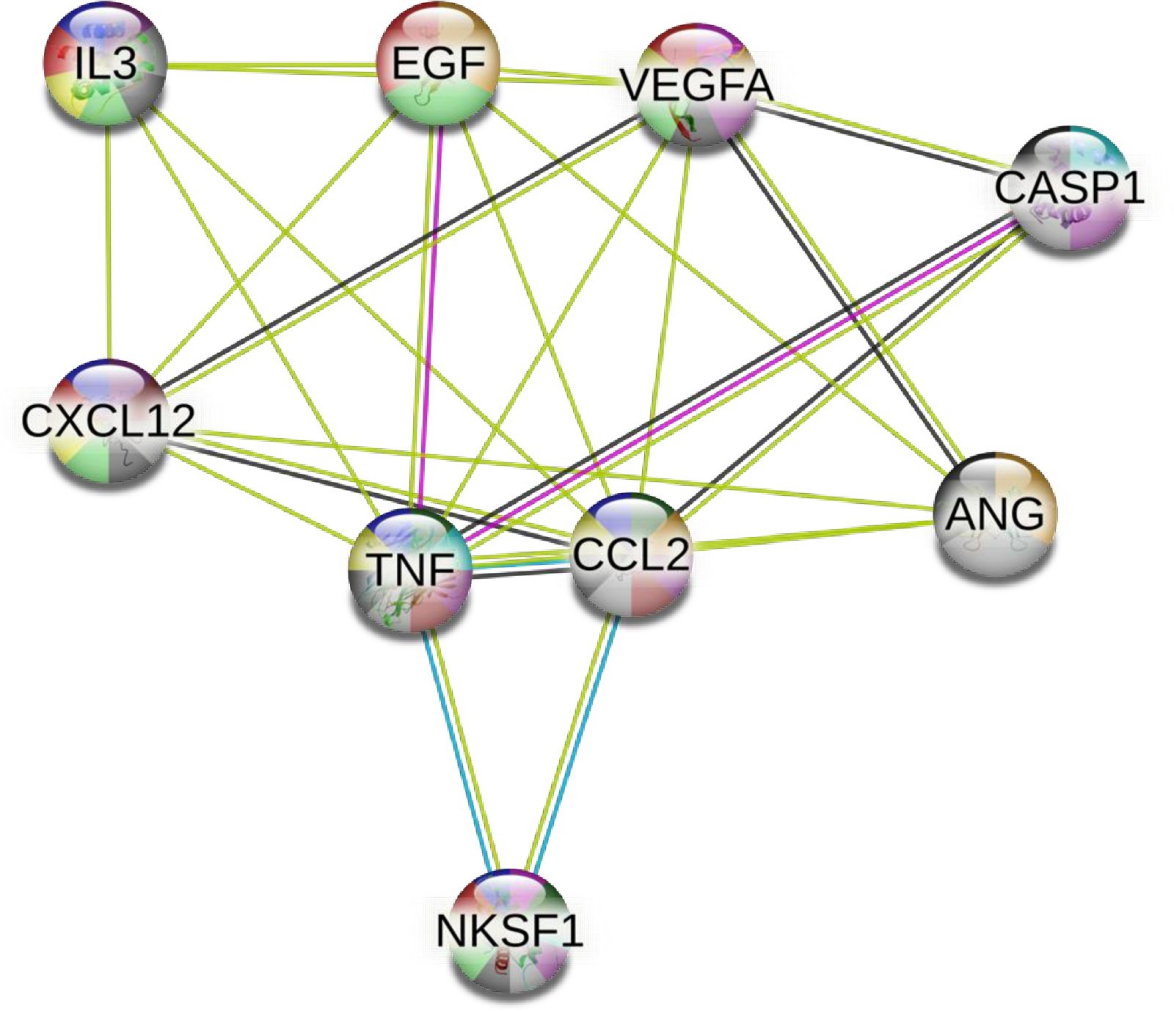
Physiological pathways and connections between cytokines and growth factors that demonstrated sex differences. Connections between nine cytokines and growth factors that demonstrated some sex differences, displayed using STRING v11.5 analysis. *Nodes* are coloured to represent different functional pathways and to identify the biomarker as either a growth factor (green), or cytokine (blue). CASP1 (IL-1β), IL-3, TNF (TNF-α), VEGFA (VEGF), CCL2 (MCP-1), EGF, ANG (Angiogenin), CXCL12 (SDF-1), and NKSF1 (IL-12) are connected using green (text mining), pink (experimentally determined), blue (from curated databases), and grey (co-expression) lines.

Relationships between responsive and detectable cytokines and growth factors were assessed using STRING v11.5 [41]. All 42 targets from the antibody array were inputted into STRING v11.5 (**Fig 3**), to detect potential relationships between all targets. Functional pathways between biomarkers were identified using the STRING v11.5 database and correspond to the color coding of the nodes. The nine biomarkers that appeared to differ between males and females were inputted into STRING v11.5 (**Fig 4**). Associations, such as protein interactions, co-expression, and experimentally determined interactions, were determined between eight of the nine cytokines and growth factors. This can be used to provide insight into which pathways may be meaningful to investigate further.

*Edges-* Text mining: these biomarkers are identified as having a significant protein interaction group in the abstracts of scientific literature within the String database; Experimentally determined: these biomarkers are identified as having a protein-protein interaction, determined experimentally, within the String data sets; From curated databases: these biomarkers are identified as having a link via the String curated database; Co-expression: these biomarkers are simultaneously expressed, within homo sapiens, in response to a stimulus.

Colour Coding of Nodes Based on Functional Pathways- Growth factor activity: Green; Cytokine activity: Yellow; Regulation of blood vessels endothelial cell proliferation involved in sprouting angiogenesis: Pink; Negative regulation of endothelial cell proliferation: Dark green; Cytokine production: Light blue; Angiogenesis: Orange; Cytokine-mediated signalling pathway: Purple; Negative regulation of immune system process: Brown; Immune response: Grey; Immune system process: Black.

**Fig 5:**
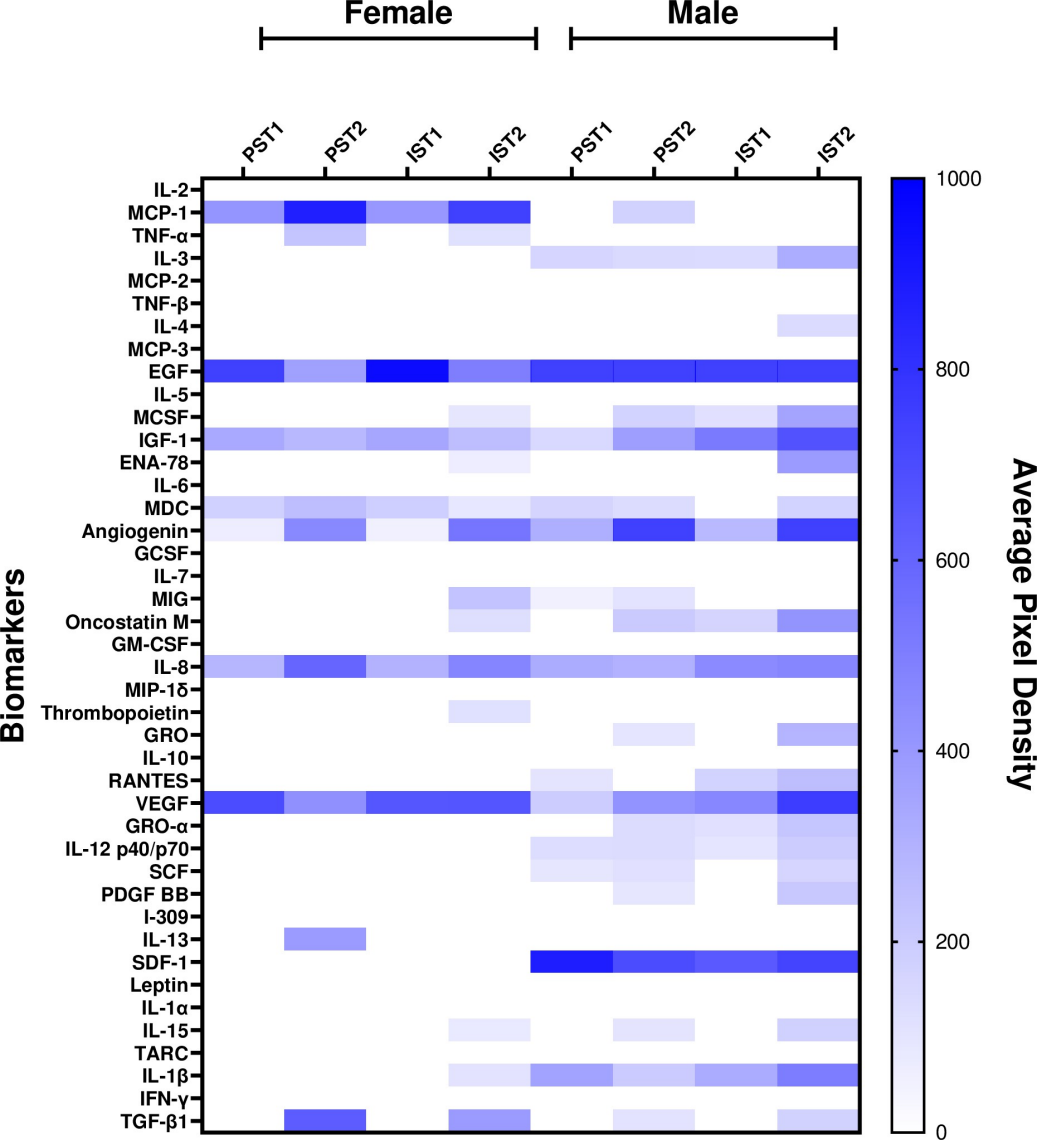
Heat map of the pixel density for the 42-Targets of the antibody array in males and females. Heat map presentation of the average pixel density for the pooled samples for males and females in each condition. Interrupted Sitting Time 1 (IS-T1), Interrupted Sitting Time 2 (IS-T2), Prolonged Sitting Time 1 (PS-T1), and Prolonged Sitting Time 2 (PS-T2). White space indicates unresponsive biomarkers for the specified condition.

Mean spot pixel densities, indicating relative levels of detectable cytokines and growth factors in saliva, from PS and IS at T1 and T2 are presented in **Fig 5** using heat maps. The heat maps indicate the relative levels of biomarkers in males and females within each condition and the potential variation in the presence of biomarkers. The heat maps clearly indicate differences in relative levels of biomarker presence in the saliva across different conditions and between sexes.

The biomarkers identified in the pooled male and females sample were then inputted into Ingenuity Pathway Analysis (IPA) [42]. Examples of two relevant pathways to sedentary physiology that indicate a clear linkage to vascular and immune responses are presented in Table 2. This table displays the number of cytokines present in each condition between males and females, and the associated pathways.

**Table 2:** Examples of pathways identified from Ingenuity Pathway Analysis (IPA) in females and males.

Pathway	Molecules							
	Females							
	PS-T1		PS-T2		IS-T1		IS-T2	
	Biomarkers	Total Number of Biomarkers	Biomarkers	Total Number of Biomarkers	Biomarkers	Total Number of Biomarkers	Biomarkers	Total Number of Biomarkers
**Atherosclerosis Signaling**	CXCL8	1	CXCL8, TGF-β1, TNF	3	CXCL8	1	CSF1, CXCL8, IL-1β, TGF-β1, TNF	5
**Chemokine Signaling**		0		0		0		0
	Males							
**Atherosclerosis Signaling**	CXCL12, CXCL8, IL-1 β	3	CSF1, CXCL12, CXCL8, IL-1β, PDGFΒ, TGF-β1	6	CSF1, CXCL12, CXCL8, IL-1β	4	CSF1, CXCL12, CXCL8, IL-1β, PDGFΒ, TGF- β1	6
**Chemokine Signaling**	CCL5, CXCL12	2	CXCL12	1	CCL5, CXCL12	2	CCL5, CXCL12	2

## Discussion

We sought to identify novel biomarkers in the saliva that are responsive to acute interventions such as our sedentary protocol. Our primary finding is that saliva provides a rich source of biomarker data that can be used to understand physiological changes in response to acute stressors or interventions. Furthermore, based on pooled male and female samples, it appears that several biomarkers may be responsive to acute interventions, and these response may vary by sex. These findings provide interesting insights into the potential use of salivary biomarkers in understanding acute response to interventions.

Our exploratory study provided us with more robust findings than we anticipated. Specifically, of the 42 targets on the microarray, 26 were detected in the pooled male and female saliva samples, including 18 cytokines and 8 growth factors. As such, we were unable to follow-up with individual sample analysis for each biomarker; however, such an analysis was conducted for IL-8 and is available elsewhere [28]. Furthermore, of the markers detected in the pooled saliva samples, many have also been detected in response to sedentary interventions in blood, such as IL-8, IL-6, IL-10, TNF-α, and RANTES [27,28,43].

Our analysis using STRING v11.5 revealed several functional pathways between the 42 targets, such as regulation of the chronic inflammatory response and negative regulation of the vascular endothelial growth factor signaling pathway. The STRING v11.5 analysis and **Figs 1 and 2** allow us to conceptualize how the biomarkers connect and work together to produce measurable immune, endothelial, and vascular responses actions. For example, STRING v11.5 identified a functional pathway between RANTES, TNF-α, IL-10, and IL-4 which builds on previous research that has shown that pro-inflammatory biomarkers TNF-α and RANTES/CCL-5 have higher concentrations with prolonged sedentary time [43–45], and have been linked to chronic diseases, including rheumatoid arthritis, inflammatory bowel disease, psoriasis, and cardiovascular disease [46–49]. Thus, the work conducted in this study lays the foundation for several lines of future inquiry using salivary biomarkers for research aimed at understanding sedentary physiology and/or sex-differences, as well as other fields assessing acute physiological responses to stressors.

Our analysis using IPA also reveals the potential to see sex differences in acute and short duration interventions. For example, IPA analysis identified that the atherosclerosis signaling pathway might be responsive to prolonged and interrupted sitting. Specifically, biomarker detection changed across the 4 conditions in females—PS-T1 (IL-8), PS-T2 (IL-8, TGF-β1, TNF), IS-T1 (IL-8), and IS-T2 (CSF-1, IL-8, IL-1β, TGF-β1, TNF)- and in males—PS-T1 (IL-8, IL-1β, CXCL12), PS-T2 (CSF-1, CXCL12, IL-8, IL-1β, PDGFB, TGF-β1), IS-T1(CSF-1, CXCL12, IL-8, IL-1β), and IS-T2 (CSF-1, CXCL12, IL-8, IL-1β, PDGFB, TGF-β1). This highlights that even in an acute study, saliva may allow us to observe sex-differences in the activation of different pathways in different conditions. The IPA software provides an interesting insight into the potential health impacts of prolonged sitting, as several pathways were noted, however, due to the exploratory nature of the work the direction of change for these pathways cannot be determined. Future work should look to confirm the change and direction of the pathways found in this study.

The heat map (**Fig 5**) shows the variation between average pixel density of the biomarkers detected in the pooled saliva samples of males and females. Overall, it appears that cytokines and growth factors were responsive to prolonged and interrupted sitting protocols, however sedentary time and sex may not be the only factors influencing salivary biomarkers. In fact, a growing body of physiological research emphasizes the need to look at individual differences when assessing the response to interventions such as exercise [50–53]. Factors such as age, sex, cardiorespiratory fitness, oral health, and more could confound changes in salivary biomarkers. As such, future work analyzing individual saliva samples should address these confounders in their analysis.

To our knowledge, this is the first study to use an antibody array-based approach to investigate a range of salivary biomarkers in the context of acute responses, particularly in the context of sedentary physiology. Previous research in the field has focused on a few well-known cytokines [54], with limited research into growth and endothelial factors. We identified eight growth and endothelial factors as well as eighteen cytokines in the saliva of healthy males and females. Thus, our work provides further support for analysis of saliva, a non-invasive and simple sampling method that can be applied across different areas of study, including sedentary and exercise physiology [55,56]. The use of the bioinformatic tools, STRING v11.5 and IPA [41,42], allowed for the determination of functional relationships between the detected biomarkers and to better understand how these biomarkers may work together to elicit measurable responses. Another strength of the current study is the homogenous sample of young, healthy individuals and the randomized cross-over design.

The major limitation of this study was the lack of individual level analysis of saliva samples. Our intention was to follow up with ELISA on markers detected using the microarray kit; however, far more markers were detected than originally anticipated making it impossible for individual level analysis on all of the markers. Nevertheless, individual level analysis was conducted using IL-8 and shows promise for use of saliva to detect changes in acute response interventions [28]. Other limitations pertaining to saliva analysis and confounders are also important to consider in interpreting the results of the present study. Previous studies have varied in the timing of their biomarker sample collection, typically occurring immediately post-intervention, 2-hours post-intervention, or 24-hours post-intervention [28,57–60]. A salivary sample collection 2-hours or 24-hours post-protocol may have allowed for the detection of other biomarkers or biomarkers to be collected at their peak of activity. Future research should consider having multiple interruption sessions with different exercise intensities for comparison. Future studies should also explore the correlation between serum and saliva samples for individual biomarkers. Regardless of validity however, it is important to note that saliva remains a valid measure [61–63] and has been shown to be an accurate method of sampling [5,6]. Finally, several confounders may influence the acute responses of salivary markers. Although we asked individuals regarding their oral health, prevalent conditions such as gingivitis or blood in the mouth could have tainted our samples. Similarly, salivary flow may have influenced salivary content. The time of collection of the samples may also influence biomarker detection as previous work has shown that cytokine profiles may be influenced by circadian rhythm and that salivary biomarkers may follow a circadian pattern [64–66]. Future work is also needed to investigate whether these factors are influenced by individual differences in addition to sex, age, and cardiovascular fitness.

In conclusion, saliva provides a promising new avenue for future research aimed at understanding acute responses to interventions in humans without the added stress of invasive procedures. Of 42 targets, 26 biomarkers were detected in the pooled saliva samples of young, healthy males and females. Future research is needed to determine how these biomarkers respond at an individual level, and what individual characteristics are associated with responsiveness of these markers.

## Supporting information

S1 Table Biomarkers investigated using the abcam human cytokine antibody array membranes. Table listing the biomarkers and their alternate name(s) investigated using the Abcam Human Cytokine Antibody Array Membranes. Biomarkers are organized by cytokine, chemokine, myokine, and growth, vascular, or endothelial factor.
(TIF)

S2 Table Average Spot Pixel Density of Cytokines (a) and Growth and Vascular/Endothelial Factors (b) to Prolonged and Interrupted Sitting Sessions at T1 and T2 in females (n = 12) and males (n = 12). Table of average spot pixel density of cytokines (a) and growth and vascular/endothelial factors (b) from both conditions, prolonged sitting (PS) and interrupted sitting (IS), pre-intervention (T1) and post-intervention (T2) for males and females.
(TIF)
