# Supplementary figures and images for: Novel biomarkers in the saliva of healthy young males and females in a randomized crossover study on sedentary time: An exploratory analysis
Source: PLoS One. 2024 Aug 20;19(8):e0308838. doi: 10.1371/journal.pone.0308838 (PMC11335159; doi:10.1371/journal.pone.0308838)

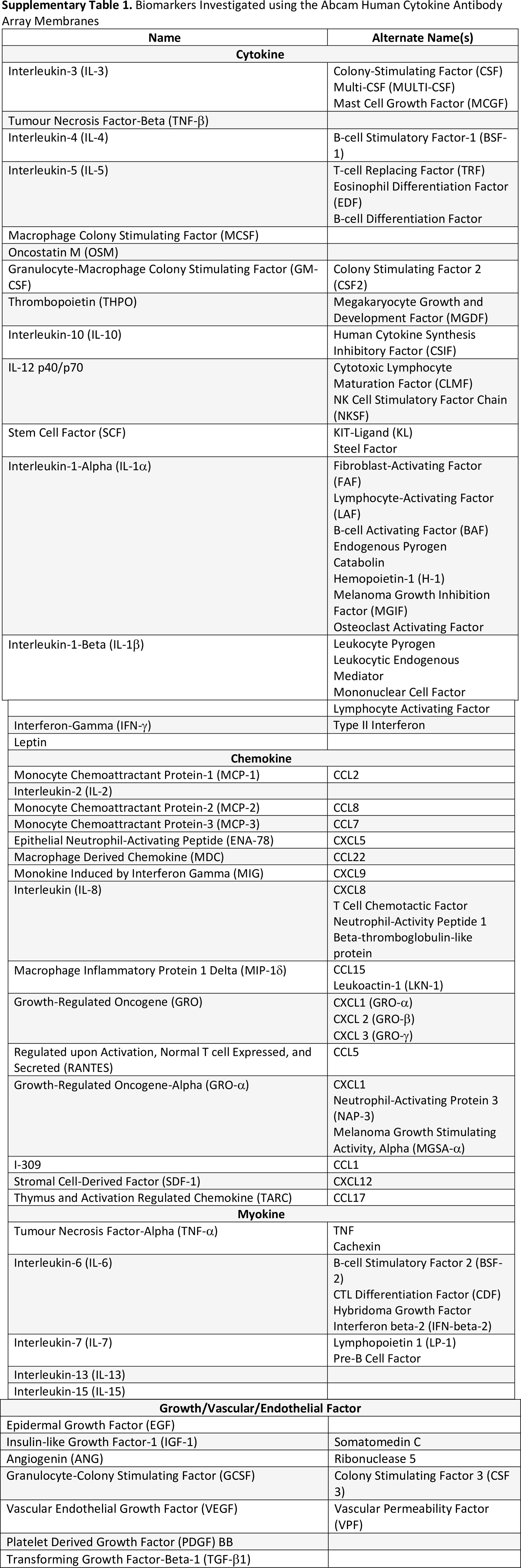

Supplement: S1 Table — Table listing the biomarkers and their alternate name(s) investigated using the Abcam Human Cytokine Antibody Array Membranes. Biomarkers are organized by cytokine, chemokine, myokine, and growth, vascular, or endothelial factor. (TIF) [file pone.0308838.s001.tif]

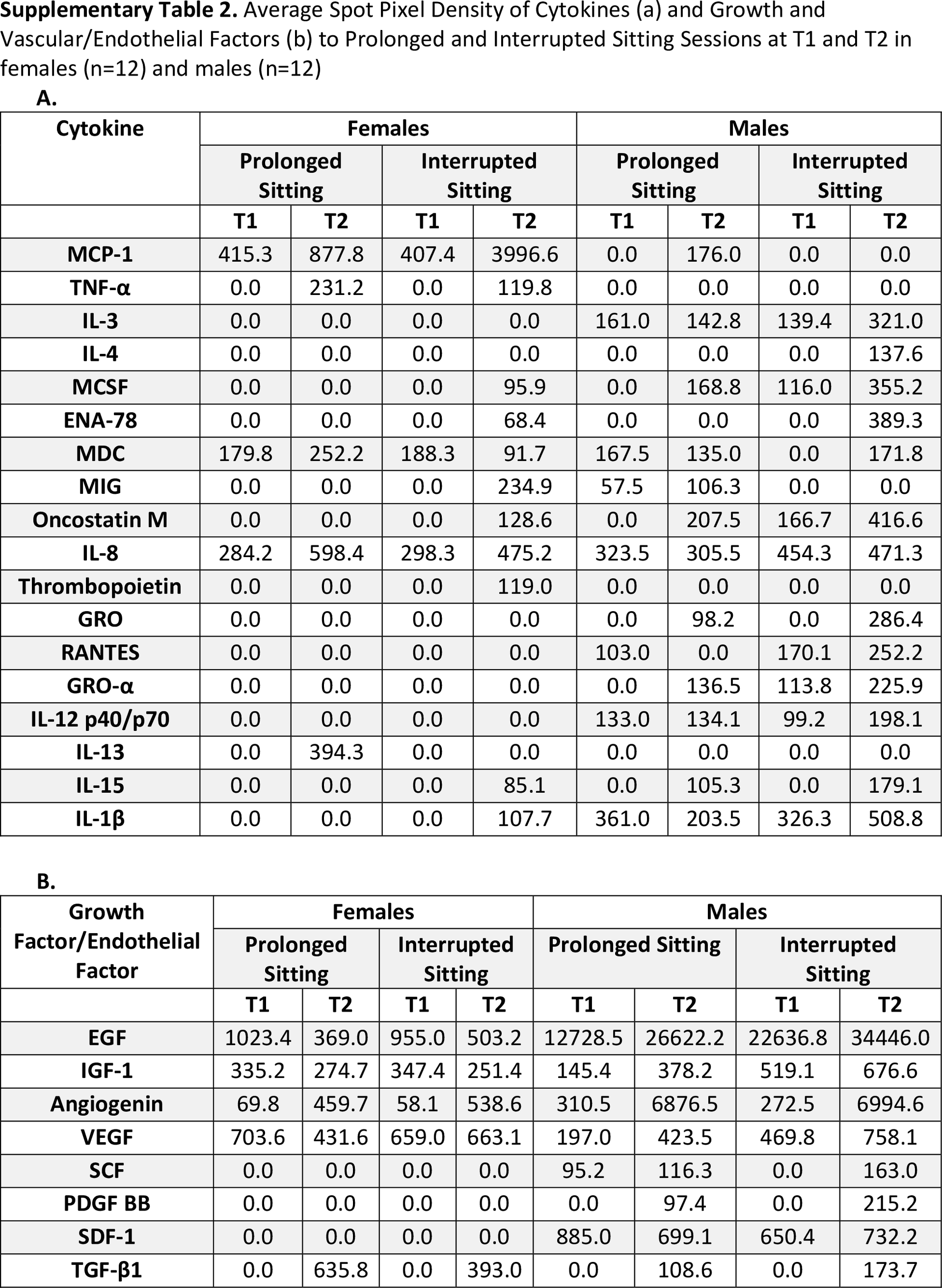

Supplement: S2 Table — Average Spot Pixel Density of Cytokines (a) and Growth and Vascular/Endothelial Factors (b) to Prolonged and Interrupted Sitting Sessions at T1 and T2 in females (n = 12) and males (n = 12). Table of average spot pixel density of cytokines (a) and growth and vascular/endothelial factors (b) from both conditions, prolonged sitting (PS) and interrupted sitting (IS), pre-intervention (T1) and post-intervention (T2) for males and females. (TIF) [file pone.0308838.s002.tif]
